# Static Guided Endodontic Approach for Pulp Canal Obliteration: A Case Report

**DOI:** 10.7759/cureus.42379

**Published:** 2023-07-24

**Authors:** Neil V Lewis, Shalini Aggarwal

**Affiliations:** 1 Conservative Dentistry and Endodontics, Dr. D. Y. Patil Dental College and Hospital, Dr. D. Y. Patil Vidyapeeth, Pune, IND

**Keywords:** pulp chamber space, digital stent, static guided endodontics, endodontic therapy, tooth luxation, pulp canal calcification

## Abstract

Traumatic injuries to the permanent dentition have deleterious sequelae if not treated adequately. In luxation injuries, it has been observed that tertiary dentin apposition may occur and can lead to calcification and closure of the pulp space. This is commonly referred to as pulp canal calcification or pulp canal obliteration. This often presents a challenge to clinicians when endodontic treatment is indicated. Static guided endodontic therapy has been advocated in such cases and has been successfully employed as a treatment strategy in recent years. This involves the design and fabrication of a digital stent, which serves as a guide for the clinician and provides a straight path to the targeted tissue site. This article reports a case of pulp canal obliteration secondary to a luxation injury sustained due to a vehicular accident. The case was treated using the static guided endodontic approach to achieve a minimal direct access to the targeted pulp chamber space.

## Introduction

Traumatic injuries to the permanent dentition have deleterious sequelae if not treated adequately. In the case of any kind of dental trauma, treatment planning depends on the type of dentition (developing or developed), location of the tooth, and the extent of tooth tissue that is lost due to the trauma. Injuries to the tooth, whether low-grade and repetitively sustained over a period of time or a one-time force causing catastrophic changes in the homeostasis of the tooth and its environment, lead to a triggering of the receptor activator of nuclear factor-kappa B (NF-κB)-ligand (RANKL), the receptor activator of NF- κB (RANK), and the receptor osteoprotegerin (OPG). This causes either a deposition or a resorption of the hard tissue. In luxation injuries, it has been observed in previous studies that the pulp chamber is susceptible to tertiary dentin apposition around the pulp space. This formation of tertiary dentin leads to calcification and closure of the pulp space, which is often referred to as pulp canal calcification (PCC) or pulp canal obliteration (PCO), and is seen in traumatic injuries to teeth. Obliteration of the pulp space could be a consequence of the molecular and physiologic ageing process or due to an accelerated response to severe trauma [1,2]. It can also occur due to a pulpal response to deep caries, restorations, and vital pulp therapy procedures [3-5]. Secondary dentin apposition in elderly patients and as an effect due to orthodontic forces have also been adversely reported [6,7]. For the endodontist, any of these root canal calcifications impede navigation, a thorough debridement, and shaping of the canal system. Additionally, locating the root canal orifice and the canals could lead to excessive loss (gouging and stripping) of the hard tissue and weakening of an infected but structurally intact tooth. These previously mentioned challenges have led to the development of guided endodontic therapy (GET). The GET technique used the alignment of cone beam computed tomography (CBCT) imaging and intra-oral surface scans to design and fabricate a three-dimensional (3D) printed stent. The stent serves as a guide to the clinician during the access opening procedure, which reduces the risk of iatrogenic errors. In cases of PCO/calcific metamorphosis that require endodontic treatment, guided endodontics is a minimally invasive technique that assists the clinician in achieving direct access to the pulpal tissue without necessitating excess removal of sound tissue.

This case report presents the management of a 19-year-old female patient (Indian) with a history of high-velocity trauma secondary to a vehicular accident 22 months prior to presentation. The patient also had a recent episode of palatal swelling and pain 45 days before the present-day visit, which was incised and drained by the resident physician. She was prescribed non-steroidal anti-inflammatory drugs (NSAIDs) and antibiotics for five days and was discharged. After regular follow-up with the resident physician, the patient’s symptoms subsided and she was asymptomatic; she was then referred to the dental hospital. After clinical examination and pulp sensibility testing, a conventional radiograph was taken to further evaluate the area of her chief complaint. The radiograph revealed fractured crown with an open apex with the maxillary left central incisor (tooth #21) and a deep restoration indicative of previously attempted endodontic treatment and PCO with the maxillary left lateral incisor (tooth #22); both teeth were associated with a large periapical radiolucency in the peri-radicular area of the maxillary left lateral incisor (tooth #22). It was decided to use GET to treat tooth #22, and surgical extraction was planned for tooth #21 due to its poor prognosis. This case report was prepared according to the PRICE 2020 guidelines [8].

## Case presentation

History

**Figure 1:**
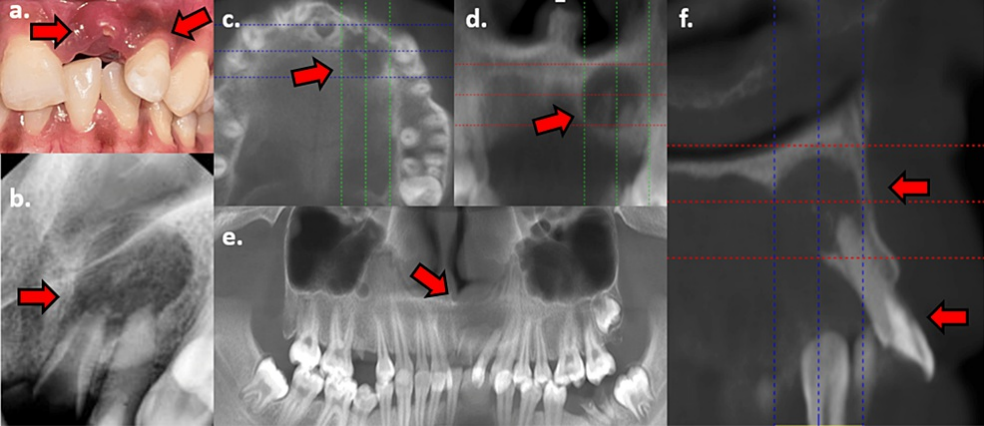
Pre-operative photograph and radiographic examination. (a) Tooth #21: grossly destructed, non-restorable, and submerged within the socket. Tooth #22: luxated with the presence of an existing restoration. (b) The 2D radiograph of tooth #21 and tooth #22 with associated periapical radiolucency. Tooth #22: existing restoration seen extending till the level of cementoenamel junction, calcified pulp chamber space seen along the long axis of the tooth; also seen are widening of periodontal ligament space and loss of lamina dura. Well-defined moderately sized periapical radiolucency is seen associated with both teeth. (c-f) Pre-operative CBCT images in axial, coronal, panoramic, and sagittal sections. Note in the sagittal section (f) the extent of the existing restoration, indicative of previously attempted endodontic treatment, and the complete obliteration of the pulp chamber space. The extent of the periapical lesion is also seen perforating the palatal bone. 2D, two-dimensional; CBCT, cone beam computed tomography

A 19-year-old Indian female patient had suffered trauma to her permanent dentition due to a vehicular accident 22 months prior to the current visit. During the time period of the vehicular trauma, the patient received treatment by medical personnel, which included soft tissue debridement and dressing. After her recovery period (two months), she sought treatment for the trauma sustained to her permanent dentition as she also experienced pain on bite pressure in the anterior region of the maxilla. The patient had then reported to the emergency medicine department 45 days prior to the present day with palatal swelling. It was unclear whether the swelling afflicted both the maxillary left central and lateral incisors (tooth #21 and tooth #22) or only one of them. It was treated as a medical emergency, and the swelling was incised and drained by the resident physician. The patient was prescribed anti-inflammatory and antibiotics for the same. After healing of the incision site, the patient was then referred to the dental wing to proceed further with treatment. On the day of reporting, the patient was asymptomatic and reported no discomfort with the area of interest. On clinical examination, tooth #21 was grossly destructed and submerged within the gingiva and alveolar socket. Tooth #22 presented with a restoration, which was placed after her initial trauma incident and was seen labially displaced due to a luxation injury as reported by the patient. The tooth was asymptomatic, and there was no observed mobility (Figure 1a).

Diagnostic procedures

**Figure 2:**
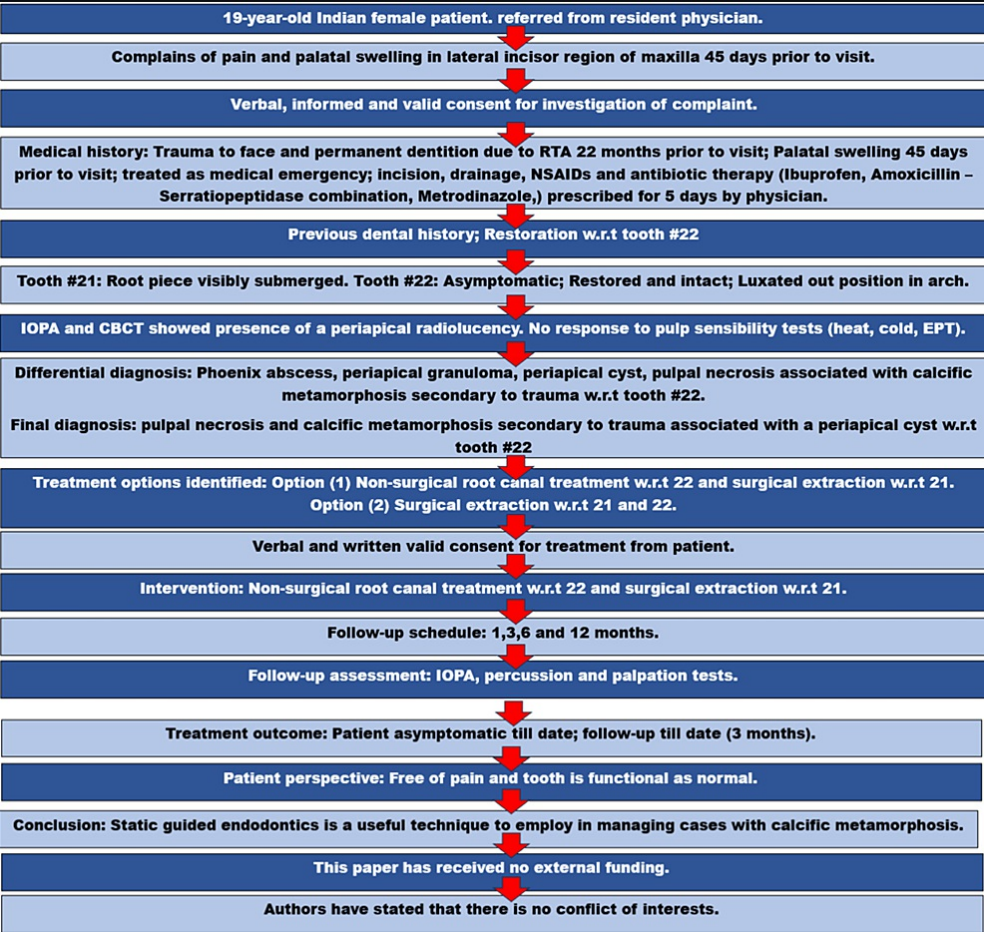
Case report prepared according to the PRICE 2020 guidelines. [8] PRICE, Preferred Reporting Items for Case reports in Endodontics

Pulp sensibility testing (thermal and electric) was performed for tooth #22, which showed no response. Radiographic (2D) examination of tooth #21 (Ellis class VIII fracture) showed coronal destruction with a large pulp space and open apex, and it was deemed unrestorable. Tooth #22 revealed a radiopaque deep restoration extending from the incisal edge to cementoenamel junction on the palatal aspect and obliteration of the pulp chamber space; both teeth were associated with a large periapical radiolucency with well-defined margins (Figure 1b). A decision to use advanced radiography (CBCT) to visualize the tooth and extent of the lesion was taken. CBCT scans (iCAT 17-19, 8.9 s, 120 kV, 5 mA) aided in visualizing the area of concern in coronal, axial, panoramic, and sagittal sections. Obliteration of the pulp chamber was seen, and the parameters of the periapical radiolucency (12 x 13 x 15 mm) were noted and further treatment was planned (Figures 1c-1f). The treatment options were identified and explained to the patient with her consent; non-surgical root canal treatment using a static guide for tooth #22 and surgical extraction of tooth #21 was chosen as the treatment of choice (Figure 2).

Static guide design and fabrication

**Figure 3:**
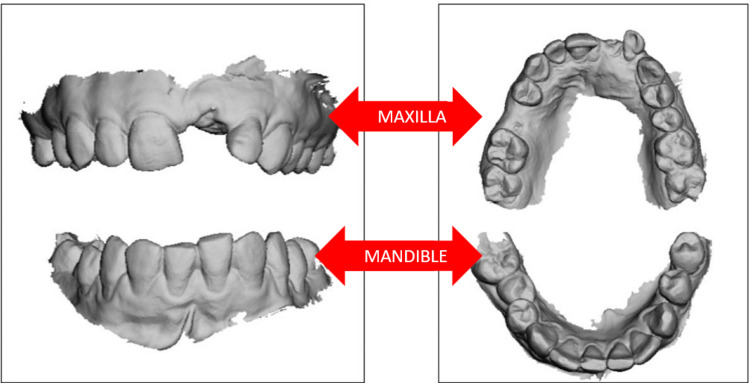
Intra-oral scans of both the maxilla and the mandible in STL format. STL, stereolithograhy

Using an intra-oral scanner (3Shape TRIOS™, 3Shape, Copenhagen, Denmark), intra-oral scans were taken and converted to STL files (stereolithography), as shown in Figure 3.

**Figure 4:**
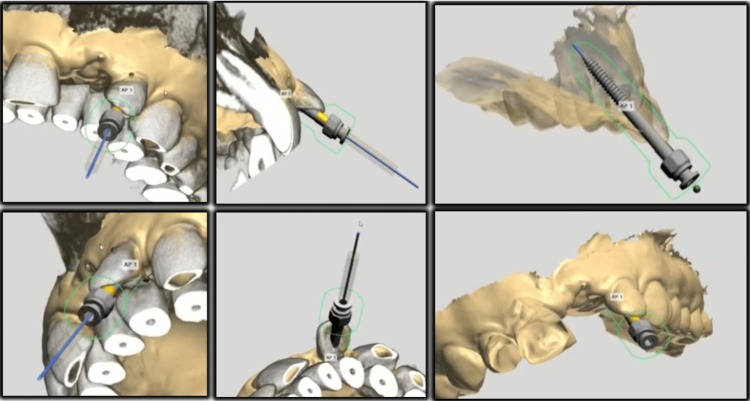
Computer-aided designing of the static guide.

Using the CBCT images, the estimated position of the accessible pulp space in the apical one-third was identified, and the minimum length required to reach the target site (17mm) was identified. Computer-aided designing (CAD) of the static guide was performed using the implant studio software (3Shape Implant Suite™), in which the images of the CBCT scans and intra-oral scans were superimposed primarily to adjust the position and fit of the guide (Figure 4).

**Figure 5:**
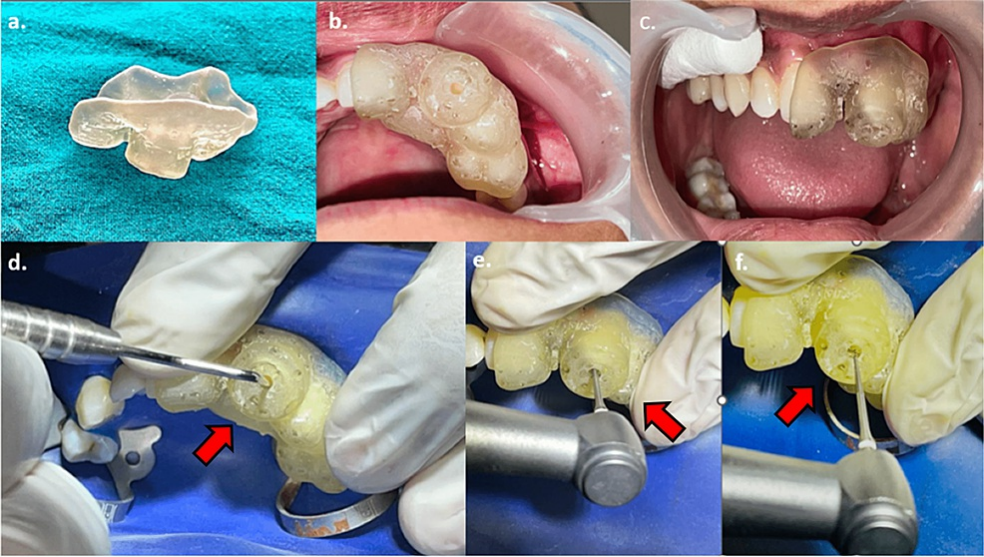
Intra-operative photographs. (a) Fabricated static guide in resin. (b, c) Checking the fit of the static guide in the oral cavity. (d) Confirming the position of the sleeve with a graduated probe. (e, f) Confirming the position of the sleeve with the access opening bur and proceeding with the access opening in a pecking motion while using water as a coolant.

An appropriately sized bur (length/diameter: 34/0.6 mm) was selected for the access opening, and the dimensions of the selected bur were noted to design the combined inner and outer sleeve dimensions of the static guide. Fabrication of the guide was done using computer-aided manufacturing (CAM) techniques; in this case, a 3D printer (3Shape) was used to fabricate the guide in resin (Figure 5a).

Treatment phase

**Figure 6:**
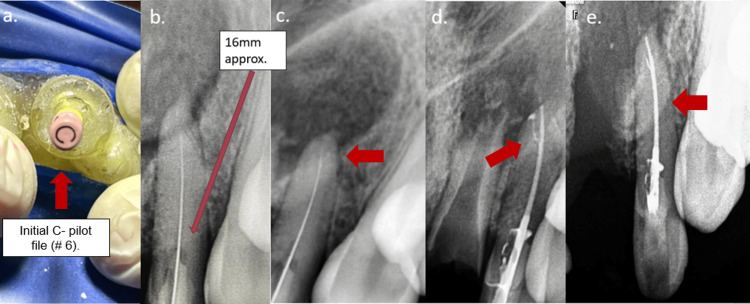
Initial filing with #6 C-Pilot file and radiographic working length determination of tooth #22. (a, b) Initial scouting done with C-Pilot file and provisional working length noted. (c) Working length determination till the radiographic apex and confirmed with apex locator. (d) Obturation done using single cone gutta-percha (#25/0.4) and bio-ceramic sealer. Note the sealing of the lateral canal at the apex with bio-ceramic sealer. (e) Follow-up at three weeks post-extraction of tooth #21.

In the next appointment, the 3D-printed guide was checked for fit in the patient’s mouth (Figures 5b, 5c). Local anesthesia (NEON Laboratories, Palghar, Maharashtra, India) with vasoconstrictor (1:200,000) was administered using local infiltration. Isolation was achieved using a rubber dam (Coltene Dental Dam Kit™, Coltene, Altstätten, Switzerland). To check and confirm the path of insertion of the bur, the tip of a graduated probe was dipped in methylene blue dye material and inserted into the sleeve space. The marked dye impression of the graduated probe was counter-checked with the insertion of the bur through the sleeve (5D). The access opening was performed in a pecking motion using a low-speed handpiece using a 34-mm-length round carbide bur (ET burs) with intermittent water spraying as a coolant (Figures 5e, 5f). During the access opening procedure, a #6 C-Pilot File was used to scout the canal. Patency was achieved at a depth of approximately 15 mm. Working length was determined, followed by cleaning and shaping protocol using hand K-files (2%) in sequential order (#6, #8, #10, #15, #20, and #25, followed by rotary files # 20 and #25; Hero Shaper Gold 4%), as shown in Figures 6a-6c.

Using a 30-gauge side vented needle, intermittent irrigation was performed using 5.25 % sodium hypochlorite with surfactant (Cerkamed, Stalowa Wola, Poland), 17% ethylenediaminetetraacetic acid (Prime Dental, Thane, Maharashtra, India), and a final rinse using 2% chlorhexidine (Prime Dental) and normal saline. The canal was dried with sterile absorbent paper points, and calcium hydroxide was applied as intracanal medicament for a period of seven days and sealed with a temporary restoration (Cavit G 3M ESPE™, Seefeld, Germany). In the second appointment, the temporary cement was removed, and the canals were irrigated with normal saline and then dried with sterile absorbent paper points. Triple antibiotic paste (ciprofloxacin, metronidazole, and doxycycline) was placed in the canals using a Lentulo spiral and sealed again with a temporary restoration for 21 days. At the third sitting, after removal of the temporary restoration, the canals were irrigated as per the protocol mentioned above and dried. The canal was then obturated using Gutta-percha (25/0.4) and Bio-ceramic sealer (Nishika Canal Sealer BG™, Nippon Shika Yakuhin, Yamaguchi, Japan) and a permanent restoration using composite resin was placed. Surgical extraction and curettage of the socket was then performed for tooth #21 using surgical extraction forceps and curettes (GDC™ Fine Crafted Dental Pvt. Ltd., Hoshiarpur, India). During the extraction of tooth #21, the periapical pathology was curetted out of the socket as a conservative approach as the lesion perforated the palatal plate of the maxilla, leaving a thin buccal plate of maxilla intact. The buccal approach would render tooth #22 inadequately supported by the bone. The patient was then recalled for follow-up at one week and three weeks. Intra-oral periapical radiographs were taken to confirm the healing process (Figures 6d-6e) and the ongoing follow-up is of three months.

## Discussion

PCO, also referred to as pulp canal calcification, calcific metamorphosis, or dystrophic calcification, is commonly associated with trauma, but other etiological factors such as caries, deep restorations, trauma from occlusion, physiologic change, and orthodontic movement have also been observed [1-7].

**Figure 7:**
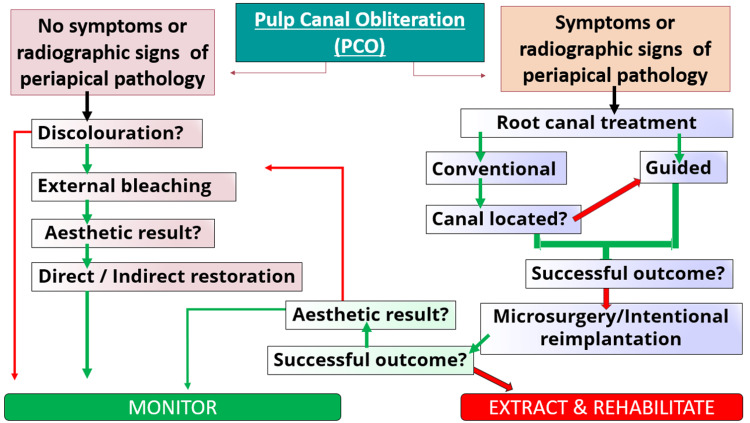
Treatment strategies for pulp canal obliteration. [9]

It is a radiographical finding, which normally presents clinically with a tooth associated with a history of trauma. They are more commonly asymptomatic, discolored, and not indicated for endodontic treatment. If aesthetics is the primary concern, it is normal to employ external bleaching techniques before internal bleaching for teeth that are asymptomatic. Evidence of radiographic changes or radiolucency present in the periapical region dictates the need for endodontic intervention. The type of intervention selected is based on the patient’s symptoms as well as the size and extent of periapical lesion (Figure 7) [9].

Due to the apposition of tertiary dentin in these cases, the irregularly calcified pulp chamber poses various challenges in achieving access to the small volume of remaining pulp tissue at the apical one-third. This often leads to iatrogenic errors such as perforations, loss of sound tooth structure, and peri-cervical dentin, which reduces prognosis of the tooth to be treated. If conventional treatment without the use of a guide fails, the economics of an implant would be greater than that of a GET protocol [10,11].

The prevalence of anatomic complexities, such as lateral canals, accessory canals, and anastomoses, makes it difficult to efficiently clean the pulp space and often requires the use of adjunctive methods (e.g. manual agitation, ultrasonic agitation, and laser activation) to improve the cleaning efficacy [12].

Advancements in the field of digital dentistry led to the recent development of GET. They are divided into static navigation (SN) and dynamic navigation (DN). The guided endodontic approach uses advanced imaging, and CAD-CAM technologies used in guided implant dentistry to design and fabricate a digital stent provide the clinician with a straight path to follow while attempting minimally invasive endodontic access to the targeted tissue site. Digital planning does require more time, but the patient-chair time is significantly reduced, and the preparation is conservative with minimal tissue loss, as the clinician sacrifices no tissue while scouting for the canal.

The SN or static GET requires CBCT and intra-oral scanner to obtain pre-operative images/scans to aid in the design of a template/guide. The resultant CBCT imaging files are saved in the Digital Imaging and Communication (DICOM) format and the intra-oral scans subsequently in the surface tessellation language (STL) format. These files are then superimposed on an image processing software to design the stent/guide whilst incorporating the path of insertion of the bur and its sleeve dimensions for a safe entry. [13] The guide can either be milled or printed depending on the systems available, after which the fit and stability should be checked before the start of treatment. The DN technique includes the use of a specifically designed mobile unit or dynamic navigation system (DNS) with an integrated software, which is used with a stereoscopic motion tracking camera, and a calibrated handpiece to provide real-time tracking of the clinical procedure until the previously determined targeted tissue site or reference point is reached. It uses a similar protocol to SN but requires the CBCT imaging files to be uploaded on the DNS planning software, which is unique to the system used, after which the handpiece is calibrated to the patient according to instructions provided by the manufacturer, as it is unique to different systems available. The clinician is then able to monitor and track the access cavity preparation through the guide during the clinical procedure [13].

The type of drill/bur used to gain access depends on availability, convenience, and operator skills. Previous studies have employed the use of twisted drills, customized drills, and long-shank round carbide burs, which are all available in various sizes according to the need of the clinician [10]. Factors that play a role in achieving access include the size and location (anterior/posterior) of the tooth, dimensions of the drill/bur, target tissue location, and guide dimensions. When the canal is located, the cleaning and shaping protocol used depends on the treatment plan decided by the clinician.

The accuracy of the drill path was checked using radiographs every 1.5-2 mm of drilling with the use of graduated probe, eventually approaching the proximity of the targeted tissue site. Previously, studies have reported the use of conventional radiographs to evaluate the accuracy of the drill path, and qualitatively they are divided into two groups: drill path centered (optimal precision) and drill path peripherally or tangentially transported (acceptable precision). From all 3D aspects, the angle of deviation reported by previous studies is 1.59-1.8° at the tip of the bur (0.12-0.47 mm). Treating mandibular incisors are more challenging due to the smaller size of the tooth and reduced margin for error, as this may cause loss of sound structure and may also result in iatrogenic errors such as perforations. Hence, the use of smaller diameter (0.85 mm) burs/drills has been recommended; in this particular case, the bur selected was 34 mm in length and 0.6 mm in diameter to ensure a minimally invasive approach considering the size of the tooth treated [14-16].

 Drawbacks and limitations

The limitations of this case report is the short follow-up period (three months) and the economics involved in the treatment such as the availability of a CAD-CAM system, and the intra-oral scanners and the burs used. Digital planning is time-consuming, and there is significant skill involved in designing the digital stent. There are also few drawbacks or concerns while performing guided endodontic treatment such as micro-crack formation that originates from the root surface during access opening and the heat generated on the external root surface that may result in injury to periodontal ligament and surrounding bone tissue. Long-shank smaller diameter burs (0.85 mm) when compared to larger diameter burs (1.5 mm) generate lesser heat; hence, in this case, a 0.6-mm size bur was used and is preferred [17,18]. The skill of the clinician is also a factor as any deviation or error may result in the fracture of the drill itself. Further long-term studies are required to properly assess and evaluate the detrimental effects of burs while using GETs. The use of advanced radiography maybe a drawback as there is a use of higher radiation dose and hence should be judiciously used. Smaller field of views can be used effectively in the treatment of PCO, therefore reducing the overall radiation exposure to the patient. In this case, the use of CBCT is indicated as it is required for the evaluation of the complex root canal anatomy according to the position statement by the 2014 European Society of Endodontology guidelines [19]. The evaluation is used in the fabrication of a digital stent in GET. Higher failure rates have been reported while using the conventional approach to treat PCO as it may lead to iatrogenic errors due to the complex root canal anatomy [20]. As reported by Kiefner et al., in cases of PCC, the conventional treatment approach is possible when treated by a specialist with the use of magnification [6]. The use of intra-oral scanners allows great accuracy when considering digital planning with 3D models. Guided endodontics is reliable when considering the treatment of PCO, and previous case reports have not reported any incidence of root perforations during GETs.

## Conclusions

Guided endodontics employs a minimally invasive endodontic approach to successively treat teeth with pulp and periapical disease. In the treatment of PCO, bypassing the laws of access cavity using a digital stent provides clinicians with a straight path to follow, which makes it easier to treat. Digital planning is time-consuming and crucial as it contributes to the ease of treatment, thus reducing the patient-chair time. Using guided access cavity allows rapid and accurate treatment while conserving tooth structure and is a viable treatment modality in cases of compromised tooth anatomy.
